# Microbial and Sensory Quality Changes in Broiler Chicken Breast Meat During Refrigerated Storage

**DOI:** 10.3390/foods13244063

**Published:** 2024-12-17

**Authors:** Anna Augustyńska-Prejsnar, Miroslava Kačániová, Paweł Hanus, Zofia Sokołowicz, Mirosław Słowiński

**Affiliations:** 1 Department of Animal Production and Poultry Products Evaluation, Institute of Food and Nutrition Technology, University of Rzeszow, 35-959 Rzeszow, Poland; aaugustynska@ur.edu.pl (A.A.-P.); zsokolowicz@ur.edu.pl (Z.S.); 2 Institute of Horticulture, Faculty of Horticulture and Landscape Engineering, Slovak University of Agri-Culture, Trieda Andreja Hlinku 2, 949 76 Nitra, Slovakia; miroslava.kacaniova@gmail.com; 3 School of Medical & Health Sciences, University of Economics and Human Sciences in Warsaw, Okopowa 59, 01-043 Warszawa, Poland; 4 Department of Food Technology and Human Nutrition, Institute of Food and Nutrition Technology, University of Rzeszow, 35-959 Rzeszow, Poland; 5 Department of Food Technology and Assessment, Institute of Food Sciences, Warsaw University of Life Sciences, Nowoursynowska 159, 02-787 Warsaw, Poland; miroslaw_slowinski@sggw.edu.pl

**Keywords:** poultry meat, refrigerated storage, microbiological quality, sensory quality

## Abstract

The aim of the study was to assess the bacterial flora of broiler chicken breast meat using the MALDI method, as well as its sensory evaluation while stored refrigerated at a stable temperature (0.5 °C+/−0.5 °C). Bacterial identification based on peptidic spectra obtained by matrix-assisted laser desorption ionisation time-of-flight (MALDI-TOF MS) mass spectrometry is a rapid, inexpensive, and accurate method for identifying isolates that belong to certain bacterial phyla. The microbiological and sensory quality was assessed on the 1st and 3rd, 5th, 7th, 8th, 9th, 10th, 11th, and 12th day of refrigerated storage. The study identified psychrophilic bacteria to be the dominant microflora during the entire period of refrigerated storage. The species profile of the bacteria, however, varied in the subsequent days of storage. From the 8th day of storage, the profile was dominated by bacteria of the genus *Pseudomonas*. The proportionate content of *Pseudomonas* bacteria ranged from 89% on day 8 to 95% on day 11th of storage. The majority of the unfavourable microflora (*Aeromonas* species, *Alcaligenes* spp., *Klebsiella* spp., and *Yersinia* spp.) were observed on the 11th day of storage, which indicates that meat spoilage processes had commenced. The quality of breast meat from broiler chickens stored at 0.5 °C+/−0.5 °C was sensorially acceptable up to the 8th day of storage.

## 1. Introduction

Broiler chicken breast meat is characterised by a high protein content (20–24%) and a significant water content (70–76%), thus making it a perishable product that quickly loses freshness during storage [1,2]. Refrigeration is a common method of preserving poultry meat [3]. The basic condition for obtaining satisfactory effects of cold storage on broiler chicken meat is to maintain a high hygienic standard during the entire pre- and post-slaughter process, high microbiological quality of carcasses, and compliance with the principle of the so-called continuity of the cold chain at the time of slaughter [4,5]. The most frequently detected microorganisms in poultry carcasses belong to the genera *Acinetobacter*, *Pseudomonas*, and the Enterobactericeae family, while *Aeromonas* spp., Micrococcaceae and Lactobacillaceae are less common [4,6,7].

The loss of freshness of broiler chicken meat stored in refrigerated conditions depends mainly on the initial microbiological load and its storage temperature [8,9,10,11,12]. A decisive factor limiting the storage of meat in refrigerated conditions is the minimum temperature of growth for microorganisms, non-specific and pathogenic inclusive [12]. When assessing the microbiological quality of refrigerated poultry meat, the total number of microorganisms and the number of bacteria of the genus *Pseudomonas* are most often taken into account [12,13,14,15]. *Pseudomonas* spp. bacteria are considered to be specific microorganisms that, through the degradation of meat proteins and the production of metabolites, cause physical and chemical changes in chicken meat, which, in turn, causes adverse sensory changes, including unpleasant smell, unacceptable colour changes, and the appearance of mucus [16,17,18,19]. Refrigerated storage of fresh meat requires a quick and effective reduction of the product temperature below 4 °C and maintaining a temperature not exceeding 4 °C throughout the entire period of storage and transport [20]. On the other hand, it is important to maintain the temperature at a constant level throughout the storage period, as temperature stability is also important for maintaining its microbiological quality and sensory characteristics [8,9,21,22]. The preservation of food products’ quality during storage is a crucial step for reducing food waste and enhancing sustainability [11].

The development and improvement of research methods, including the MALDI-TOF MS (matrix-assisted laser desorption ionisation time-of-flight mass spectrometry) method, has made it possible not only to quantify the microbiological assessment of stored meat, but also to assess the profile of the meat microflora [23,24]. The matrix-assisted laser desorption/ionisation time-of-flight mass spectrometry (MALDI TOF MS) method is based on the analysis of the protein profile of the organism. The identification of microorganisms is based primarily on the detection of ribosomal proteins, but also mitochondrial proteins that can be isolated [23,24,25]. This method has found its special place in food microbiology, as a fast and inexpensive method, additionally characterised by high accuracy in identifying bacteria [25]. An important advantage of the method is also the small amount of material required for analysis, i.e., one bacterial colony [24]. Assessment of the microflora profile of broiler chicken breast meat may have both knowledge and application significance for improving the methods and conditions of its storage [25,26].

The quality of raw broiler chicken meat is determined by its positive sensory evaluation [27] as it is associated with consumer acceptance during purchase and during the preparation of meat for consumption [28]. Cooling and maintaining a stable refrigeration temperature throughout the storage period reduces the growth and multiplication of microorganisms and slows down the intensity of chemical transformations associated with the deterioration of the sensory quality of meat [29,30,31].

The aim of the study was to assess the bacterial flora of broiler chicken breast meat using the MALDI-TOF MS [matrix-assisted laser desorption ionisation time-of-flight] method, as well as the sensory evaluation of meat during storage under stable cold temperature (0.5 °C+/−0.5 °C).

## 2. Materials and Methods

### 2.1. Research Material

The raw material for the study was the breast meat of broiler chickens obtained under production conditions in a local poultry slaughterhouse located in the Podkarpacie region of Poland. All breast muscles were obtained from 40-day-old ROSS 308 broiler chickens reared in the same flock, with a stocking density of 33 kg/m^2^ and delivered to the slaughterhouse in a specialised vehicle adapted for the transportation of broiler chickens. The slaughtering of the birds, including the butchering of the carcass and shearing of the pectoral muscles, was carried out in controlled production conditions. After chilling, 180 pieces of weight-balanced (250 ± 50 g) individual pectoral muscles not hampered with any defects were randomly collected. While maintaining sterility, they were randomly assigned to 9 study groups of 20 pieces each. Chicken breast fillets were placed in E2-type meat storage crates lined with foil bags. The foil bags and crates were approved for contact with food. The meat was stored in production conditions in a refrigerated warehouse at a stable temperature of 0.5 °C+/−0.5 °C. Temperature stability in the refrigerated warehouse was maintained using a cooling system operating in a continuous mode with temperature monitoring. Temperature monitoring in the refrigerated warehouse was carried out using specialised temperature sensors (SIMEX RS-485, SIMEX, Gdańsk, Poland) connected and configured with the SimCorder automatic temperature measurement software (SimCorder version 4, SIMEX, Gdańsk, Poland), with an accuracy of 0.1 °C and recording every 15 min. Microbiological and sensory quality was assessed on the 1st (24 h after slaughter) and 3rd, 5th, 7th, 8th, 9th, 10th, 11th, and 12th days of refrigerated storage. To achieve this objective, one randomly selected container containing 20 pieces of pectoral meat was transported at a temperature of 0.5 °C+/−0.5 °C to the Laboratory for the Evaluation of Poultry Products, University of Rzeszów on each day of the assessment. In the laboratory, while one research team immediately undertook the meat’s microbiological assessment, the other performed the sensory evaluation of the meat.

### 2.2. Microbiological Analysis

The preparation of the breast muscle samples for storage is described in detail in Section 2.1. Research Material. For microbiological evaluation, all breast muscles were stored in E2-type meat storage containers lined with food-grade film. On each day of the study, 10 breast muscles were allocated for microbiological analysis, from which 10 replicates were randomly taken. Using sterile scalpels and forceps, 5 g of chicken meat was removed. It was then quickly transferred into a sterile Stomacher bag with 45 mL of 0.1% buffered peptone water (BPW, pH 7.0, Basingstoke, UK) and homogenised for 60 s in the Stomacher at room temperature. For each sample, appropriate serial decimal dilutions in 0.1% BPW solution were performed. The surface of the dry medium was covered with 0.1 mL of serial dilutions of the prepared samples. After two days of incubation at 30 °C, total viable counts (TVC) were counted on Tryptone Soya Agar (TSA, Oxoid, Basingstoke, UK), and after 24–48 h of incubation at 37 °C, total viable counts (TVC) were counted on Endo Agar (EA, Oxoid, Basingstoke, UK) the number of Enterobacteriaceae was counted, and after 48–72 h of incubation at 25 °C the number of *Pseudomonas* genera was counted on *Pseudomonas* agar supplemented with CFC/CN (PA, Oxoid, Basingstoke, UK).

After incubation, the bacterial biomass from the microbial medium was transferred to 300 μL of distilled water. Then, 900 μL of ethanol was added and mixed using Votrex (Vortex Classic, Velp Scientifica, Usmate, Italy). The mixture was centrifuged (MPW-150R, MPW MED. INSTRUMENTS, Warsaw, Poland) at 14,000 rpm (RCF = 22,570× *g*) for two minutes. The pellet was centrifuged once more after the supernatant was discarded. After pipetting off all remaining ethanol, the precipitate was allowed to dry at ambient temperature. The pellet was then combined with 30 µL of 70% formic acid using a paddle. Then, 30 µL of acetonitrile was added and mixed thoroughly. After centrifuging the solution for two minutes at maximum speed, 1.5 μL of supernatant was applied to a polished MALDI target plate (Bruker Daltonics, Bremen, Germany). Immediately after drying, 1.5 µL of matrix solution was added to each spot and allowed to air dry. Samples on a polished MALDI target plate were dried at room temperature (19 °C–21 °C), which took about 15–20 min. The metal plate with the overlaid samples was placed in the measuring chamber of the apparatus; the air was then removed to create a vacuum and the plate was exposed to a laser beam. The spectra were generated by MALDI-TOF and analysed with a Microflex LT (Bruker Daltonics, Germany) instrument using Flex Control 3.4 software and Biotyper Realtime Classification v3.1. An average of 40 laser shots taken in automatic mode using the lowest laser power required to ionise the samples was used to produce each spectrum. A real-time program, Classification v3.1 (Bruker Daltonics, Bremen, Germany) was used to evaluate the spectra and compare the measurement results to the database. The manufacturer specified that successful identification must be within a confidence score of ≥2.0 for the species level and ≥1.7 for the genus level [26].

### 2.3. Sensory Evaluation

The sensory attributes of raw breast muscles stored refrigerated were evaluated by a trained 10-person panel with proven sensory sensitivity (defined on the basis of the PN-EN ISO 8586 standard [32]. Sensory panellists were previously recruited from among researchers working at the Institute of Food Technology and Nutrition of the University of Rzeszów. The panel had previous experience in sensory evaluation of poultry meat and cold-stored products. Prior to the main study, the sensory panellists were calibrated in a pre-test. During the pre-test, the panellists were introduced to the identification of sensory attributes required to describe the aroma, colour, and texture of the overall acceptability of broiler chicken breast meat during storage. Prior to the evaluation, the panelists were briefed with the evaluation questionnaire. For purposes of evaluation, 10 cube-shaped samples measuring 2 cm × 2 cm × 2 cm were cut from each pectoral meat piece, which were subsequently coded. Following this initial preparation, the samples were presented to evaluators in white containers with a transparent lids, which were placed on white trays [27]. The procedure was the same for each sample. Each panellist received 10 samples from each study group on the day of the evaluation, which were given at random. Each of the panelists received 10 samples from each study group on the day of the evaluation. The test was carried out in a duly prepared room with appropriate lighting, room temperature, free from foreign smells, as well as any distracting factors, in accordance with the PN-EN ISO 8589 standard [33]. The attributes of the assessment were: smell, external colour, consistency, and general appearance. Sensory assessment was performed using a 5-point hedonic scale according to the specifications in Table 1.

### 2.4. Statistical Analysis

The results of the study are presented as means and standard deviations. The effect of the storage duration on the microbiological parameters of the broiler chicken meat was assessed using unidirectional analysis of variance (using post hoc and Tukey’s HSD assays), while that of sensory characteristics was achieved using nonparametric Kruskal–Wallis assays. The differences were considered significant at *p* < 0.05. The calculation was performed using the Statistica 13.3 software package [34].

## 3. Results and Discussion

Cold conditions are common throughout the broiler chicken processing and distribution chain. While chill line controls used during butchering increase microbiota selection pressure, chilling remains one of the best ways to reduce microbial contamination of chicken carcasses during processing [4,5,35]. Besides cooling the carcass, another important factor in the process of obtaining meat from broiler chickens, which affects its final quality, is cooling the meat to a temperature not exceeding 4 °C [8,36]. Storage temperature has a significant impact on the growth rate of microorganisms, especially when it fluctuates [9]. The total number of microorganisms ranged from 2.34 on the first day to 6.79 log CFU/g in twelve days (Table 2). The TVC indicate several types of bacterial growth during storage, which varied between samples. This corroborates previous studies which showed that the range of total viable bacterial counts from chicken sampled after two-thirds of its storage time at 4 °C was 3 to 8 log CFU/g [37]. There was a significant association between storage temperatures and total viable counts.

The number of Enterobacteriaceae genera ranged from <1.00 on the first day to 5.56 on the last day of the study (Table 2). In an investigation conducted by Balamatsia et al. [38], the *Pseudomonas* count in air-packed chicken samples reached approximately 7.0 log cfu/g after approximately 8 days of storage. *E. coli* is the most commonly used indicator of faecal contamination of food [39]. Its counts usually correlate more closely with those of Enterobacteriaceae, which are commonly associated with elevated counts on poultry carcasses due to mishandling, improper or unsanitary processing, and/or storage conditions [40]. Faecal contamination of beef and chicken meat with Enterobacteriaceae, including *Salmonella* spp., *E. coli*, *Proteus* spp. and *Klebsiella* spp., is a significant food hygiene problem [41,42]. Effective monitoring of the presence and accurate identification of zoonotic bacterial pathogens in food is critical to minimising the incidence of foodborne illness and reducing microbial contamination of food.

The current study revealed that *Pseudomonas* counts ranged from 1.76 on the first day to 6.62 log CFU/g (Table 2). In the study conducted, the deterioration in sensory characteristics, mainly meat odour during storage, was strongly correlated with an increase in the number of *Pseudomonas* spp. bacteria, which were the predominant bacteria present in the meat evaluated (Table S1). The metabolic activity of *Pseudomonas* ssp. bacteria during aerobic refrigerated storage results in the formation of metabolites, sensorially perceptible as unpleasant odour [2,21]. According to Franke et al. [43], *Pseudomonas* spp. are commonly responsible for the spoilage of chicken meat stored under aerobic packaging conditions. At the end of the meat’s shelf life, *Pseudomonas* were not only found as dominating, but contributed to meat spoils through proteolytic, lipolytic, saccharyllytic, and biosurfactant processes [14]. The action of these microorganisms consists in the enzymatic acceleration of protein proteolysis as well as the oxidative and hydrolytic processes of tissue fats [16,44]. It is worthy of note that 7–8 log CFU/g for *Pseudomonas* spp. is a factor that determines the putrefaction of fresh meat according to Nychas et al. [45]. In an investigation conducted by Balamatsia et al. [38], *Pseudomonas* counts in air-packed chicken samples reached approximately 7.0 log CFU/g after approximately 8 days of storage. Another study found that at 2 °C, total Enterobacteriaceae and *Pseudomonas* spp. counts in meat increased over time and reached 4.64 log CFU/g, 4.16 log CFU/g, and 4.48 log CFU/g, respectively, on the eighth day of cold storage. The rate of bacterial growth in meat stored at 6 °C, in each of the evaluated periods, was higher than in meat stored at 2 °C [26].

**Figure 1:**
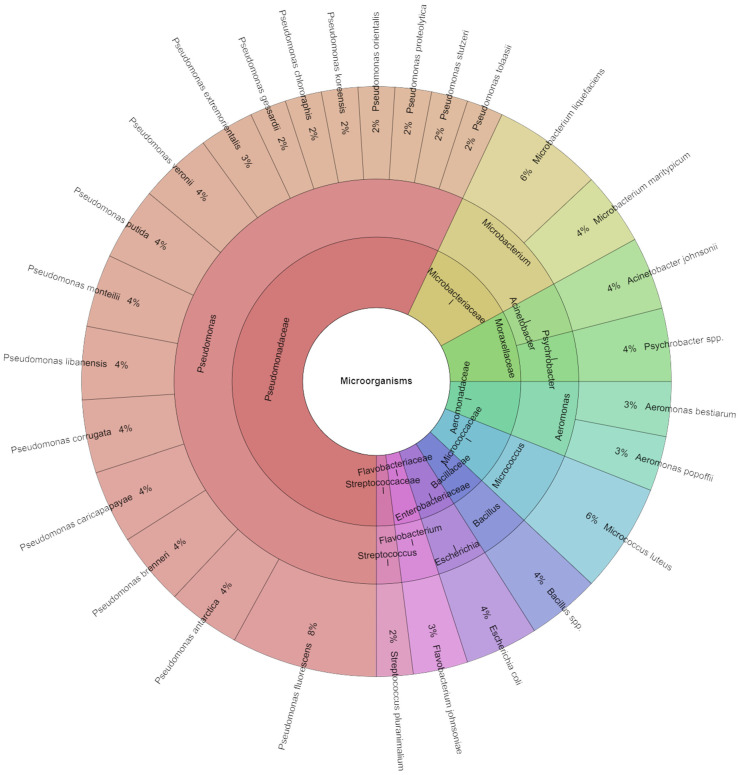
Krona chart: Isolated species from day 1.

In total, 48 isolates were identified from meat samples on the first day (Figure 1). On the first day, samples of 10 families, 10 genera, and 28 species were isolated. The most isolated species in day one were *Pseudomonas fluorescens* [8%], followed by *Microbacterium liquefaciens* and *Micrococcus luteus* [6%]. The species *Pseudomonas fragi*, *Pseudomonas lundensis*, and *Pseudomonas fluorescens* are the most common pseudomonads found in poultry meat [46,47]. The primary genera of Enterobacteriaceae isolated are *Serratia* (*Serratia fonticola*, *Serratia grimesii*, *Serratia liquefaciens*, *Serratia proteamaculans*, and *Serratia quinivorans*), *Hafnia* (*Hafnia alvei*, *Hafnia paralvei*), *Rahnella*, *Yersinia*, and *Buttiauxella* [48]. Several novel species of *Enterococcus* or *Lactobacillus*, including *Enterococcus viikkiensis*, *Enterococcus saigonensis*, and *Lactobacillus oligofermentans*, have also been identified in poultry meat products [49,50,51]. *Brochothrix thermosphacta* has also been frequently observed in poultry meat. Some of the numerous reports that can be found in the literature concentrated more explicitly on rotting bacteria, while others were more pathogen-oriented.

**Figure 2:**
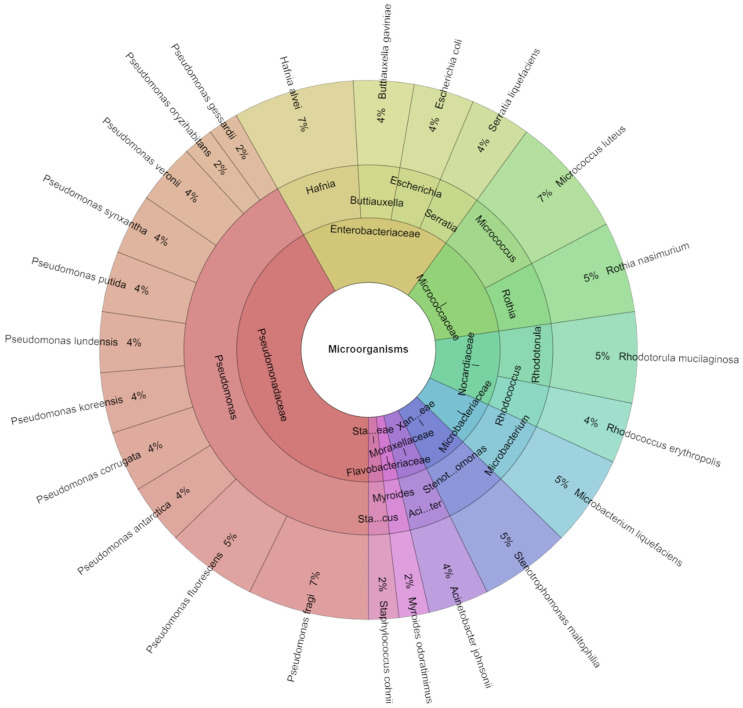
Krona chart: Isolated species from day 3.

On day 3, the number of isolates with identification at good level was 55 (Figure 2). On the third day of refrigerated storage, 10 families, 12 genera, and 24 species were identified in chicken meat. The most isolated species on day two were *Hafnia alvei*, *Pseudomonas fragi*, and *Micrococcus luteus* with 7%. *P. fragi* in cleaned non-contaminated samples and unknown bacteria in unwashed non-contaminated samples accounted for the majority of the factors. It has been demonstrated that *P. fragi*, a psychrotrophic bacterium, plays a major role in meat spoilage [52]. It is frequently discovered on fresh and aerobically damaged meat, even during storage at low temperatures [53,54].

**Figure 3:**
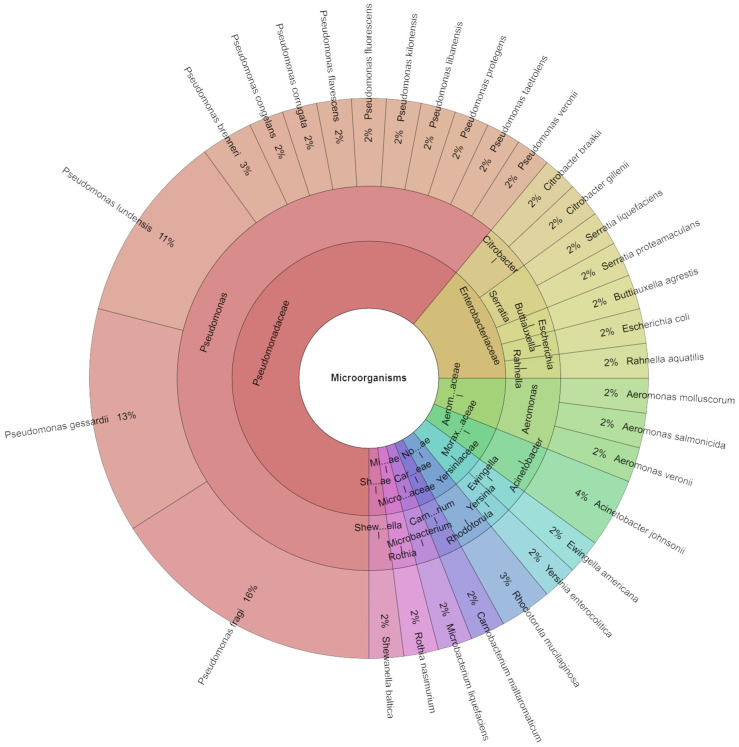
Krona chart: Isolated species from day 5.

A total of 63 isolates were identified on day 5 (Figure 3). Isolates from meat samples included 11 families, 15 genera, and 33 species with the most commonly isolated being *P. fragi* (16%), followed by *P. gessardi* (13%), and *P. lundensis* (11%). Since the psychrotrophic microorganism *Pseudomonas* causes surface change, it is a very important indicator of food spoiling in items that are stored at low temperatures [55].

**Figure 4:**
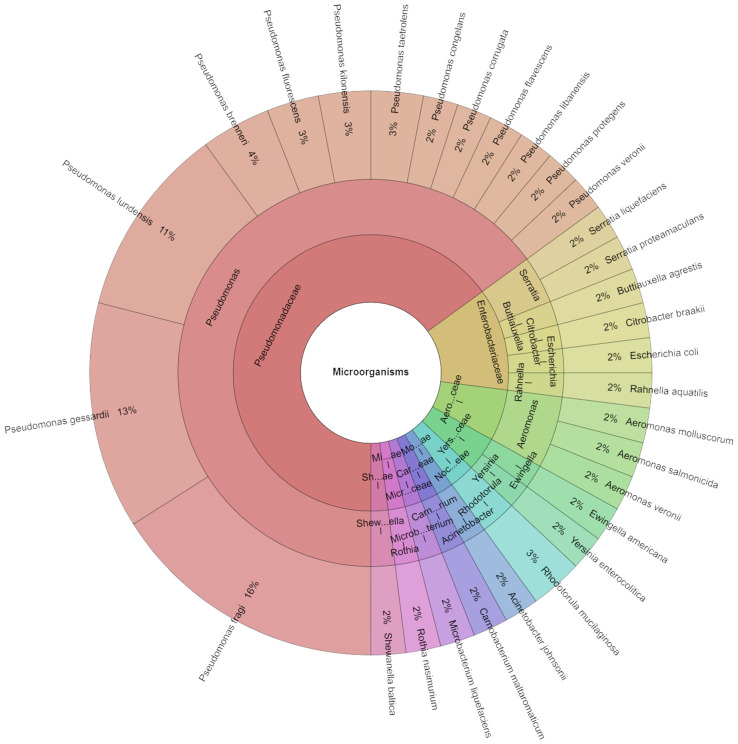
Krona chart: Isolated species from day 7.

The same tendency as in day 5 was found on day 7, where the most isolated species were *P. fragi* (16%), followed by *P. gessardi* (13%), and *P. lundensis* (11%). A total of 11 families, 15 genera, and 30 species were identified on the seventh day (Figure 4). The study identified 62 isolates with successful scores. In comparison to what was initially present, the microbiota diversity is reported to decline after storage while the bacterial load rises. [56,57]. The proliferation and metabolic activity of rotting bacteria lead to microbial deterioration. While the majority of research has concluded that the predominating bacteria in damaged food are the ones that cause spoiling, other studies have defined spoilage using the microbiological acceptability criterion (TVC of 7 log CFU/g) [57,58].

**Figure 5:**
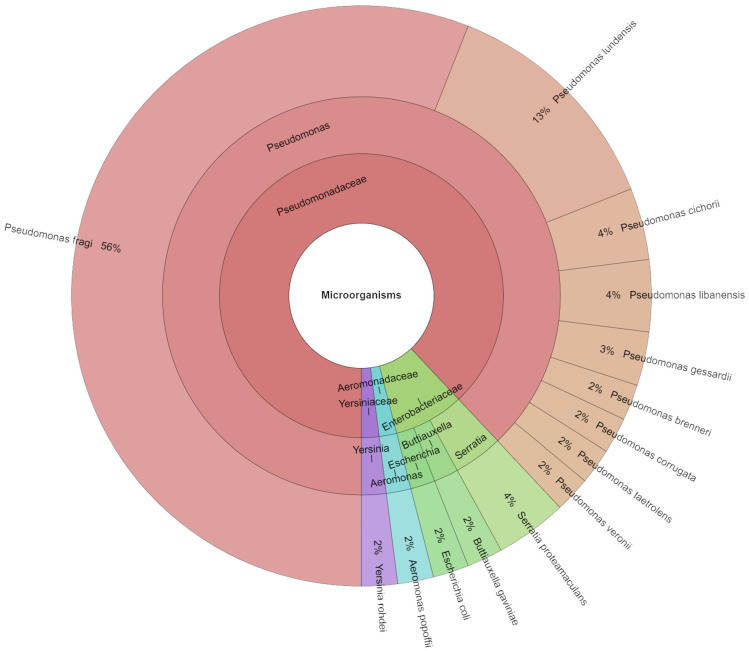
Krona chart: Isolated species from day 8.

Making use of the mass spectrometry with high score 52 isolates, 4 families, 6 genera, and 14 species were identified on day 8 (Figure 5). The most isolated species from chicken meat on day 8 was *P. fragi* with 56%, followed by *P. lundensis* with 13%. By using MALDI-TOF MS, spoilage microbes were identified. The primary organisms discovered after eight days at 4 °C and 10 °C were *Brochothrix thermosphacta*, *Carnobacterium* spp., and *Pseudomonas* spp. The primary spoilage microbiota was represented by the species *Hafnia alvei* at 10 °C and the genera *Carnobacterium* spp., *Serratia* spp., and *Yersinia* spp. at 4 °C [57].

**Figure 6:**
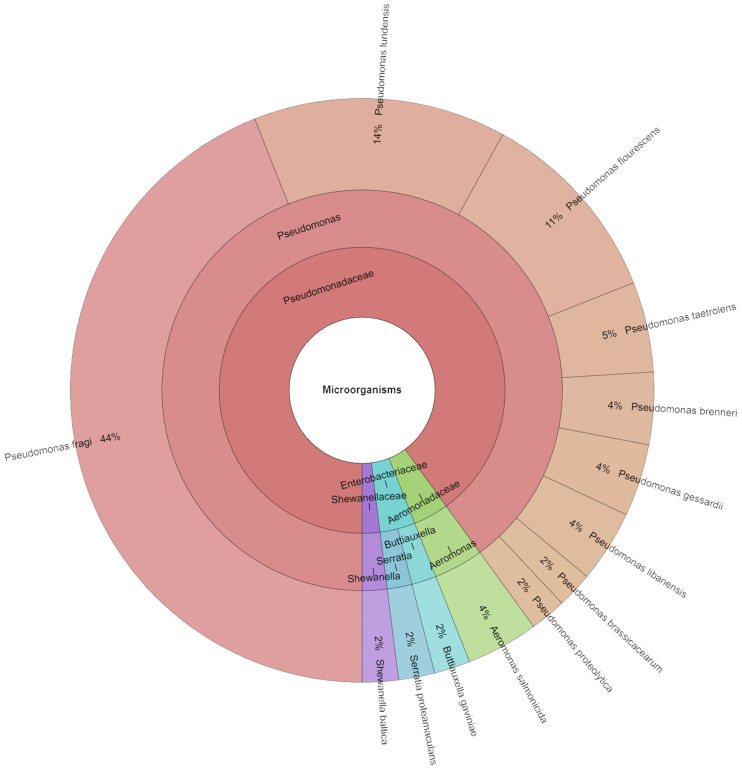
Krona chart: Isolated species from day 9.

From the meat samples on day 9, 55 isolates were identified (Figure 6). Overall, 4 families, 5 genera, and 13 species of bacteria were isolated. The most identified species on day 9 were *P. fragi* with 44%, *P. lundensis* with 14% and *P. fluorescens* with 11%. *Pseudomonas* spp. and other psychrophilic and psychrotrophic bacteria grow more readily when stored at low temperatures. Certain organisms are able to endure the entire process. An example of such species is *Shewanella putrefaciens*, which is commonly detected on carcasses during the slaughtering process and persists even after being stored in aerated places for 14 days [7].

**Figure 7:**
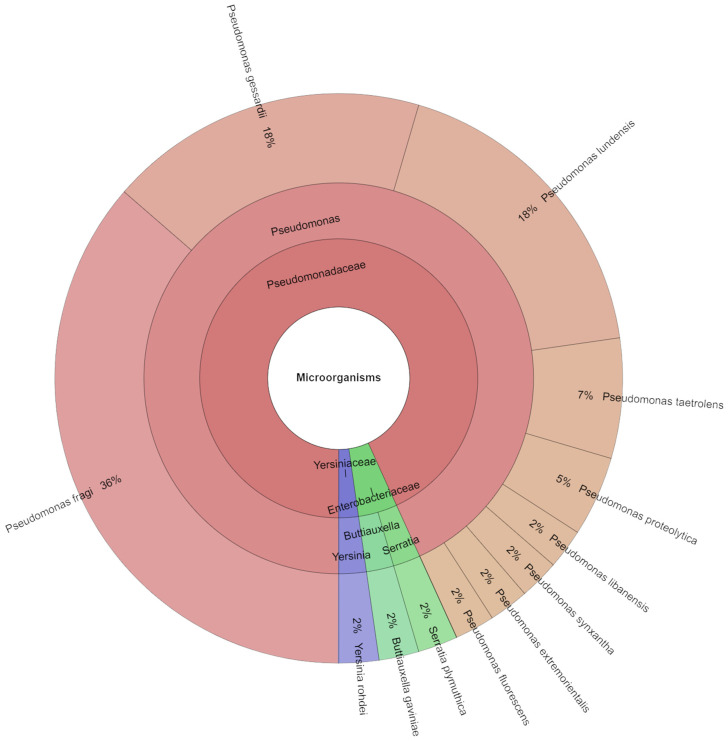
Krona chart: Isolated species from day 10.

On the seventh day, 44 isolates were found in meat samples (Figure 7). In a single day, 12 species, 4 genera, and 3 families were isolated from the samples. *P. fragi* (36%) was the most isolated species on the seventh day, followed by *P. gessardii* and *P. ludensis* (18%). It is commonly believed that spoiling is exclusively caused by a small number of representative species, also referred to as the autochthonous microbiota, which arise from the original microbial association [13].

**Figure 8:**
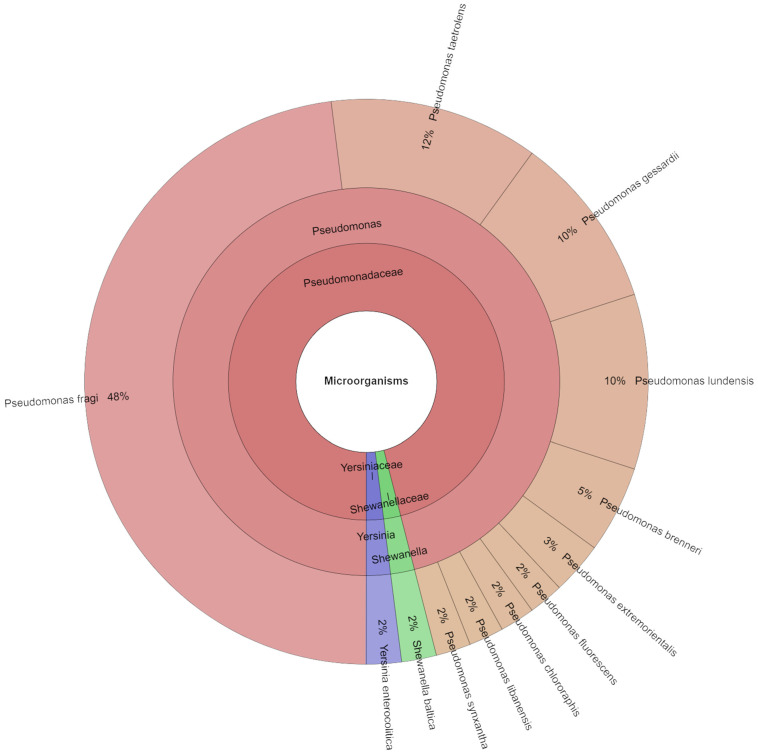
Krona chart: Isolated species from day 11.

Using meat samples, 42 isolates were found in all on the eleventh day. Three families, three genera, and twelfth species were separated from the samples on the eleventh day (Figure 8). *P. fragi* (48%) and *P. taetrolens* (12%) were the next most isolated species in the eleventh day, with *P. gessardii* and *P. lundensis* coming in at 10%. *Pseudomonas* spp. is one of the most common genera at aerobic storage, according to a number of studies. *P. fragi*, the dominant species in this group, is also found in meat that has been packaged in a modified atmosphere [13,59].

**Figure 9:**
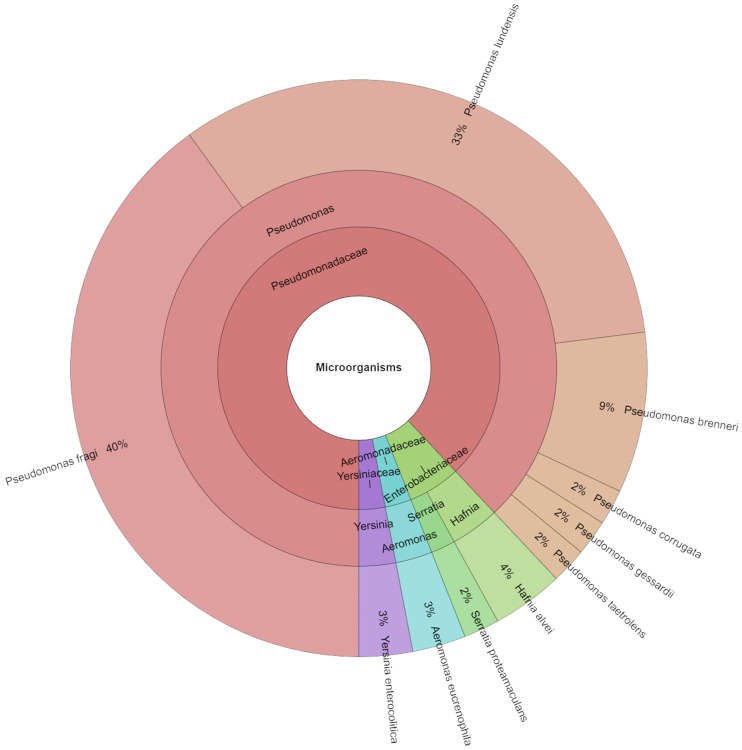
Krona chart: Isolated species from day 12.

On day twelve, forty-five isolates were found in the meat samples. Ten species, five genera, and five families of bacteria were identified (Figure 9). On the ninth day, *P. fragi* (40%), *P. lundensis* (33%), and *P. brenneri* (9%) were the most frequently recognised species. All the isolated species from all days are shown Table 3. The MALDI-TOF MS Biotyper was used to identify the meat and confirmed that culture tests revealed changes in the bacterial microflora’s composition throughout the preservation period. Psychrophilic bacteria were the predominant microflora, irrespective of the temperature used, as they are characterised by their ability to flourish in chilled circumstances. Nevertheless, it was noted that the profile of the detected bacteria altered after storage at 6 °C, with the majority of unfavourable microflora emerging, suggestive of ongoing spoiling processes. *Aeromonas* spp., *Alcaligenes* spp., *Klebsiella* spp., and *Yersinia* spp. are a few possible examples of these bacteria [60,61].

The sensory characteristics of poultry products are crucial for determining their quality and have a major impact on consumer choices [27]. The metabolic activity of microbes results in the formation of metabolites that cause physical and chemical changes in poultry meat, perceived sensorially as unpleasant smell, discoloration, and mucus [17,18]. The effect of refrigerated storage conditions on the sensory characteristics of poultry meat was presented in the studies by Augustyńska-Prejsnar et al. [26], Yimenu et al. [62], Sujiwo et al. [21], Kondratowicz et al. [44], and Garavito et al. [63]. The research findings showed that all the tested sensory characteristics of meat deteriorated with the time of refrigerated storage (Table 4). It was indicated that meat stored at 0.5 °C+/−0.5 °C was of acceptable quality until the 8th day of storage (Table 4). Significant changes in smell took place on the 10th day of meat storage. Similarly, in Katiyo et al. [2], the smell of chicken meat deteriorated faster than the colour and general appearance and was correlated with an increase in the number of microorganisms. Also, in a study by Sujiwo et al. [21] on 12 days refrigerated storage (temp. 4˚C) of broiler chicken breasts packed on polystyrene trays wrapped in low-density polyethylene, odour and colour changed faster than texture and overall acceptability. The unpleasant odour is related to the oxidation of lipids in the meat. In addition, protein degradation products released by microorganisms also lead to an unpleasant odour, so meat spoilage can be determined by an unusual odour [21,64]. Our own study showed that on the 11th day of storage, the meat odour was found to be unacceptable (altered, putrid), the colour undesirable, altered in places, with external infiltration, the texture unacceptable, with muscle tissue loosening and flattening after pressure, and an overall undesirable appearance. Changes in the deterioration in sensory characteristics were observed more rapidly compared to the results of quantitative microbiological assessment. Different results during the cold storage of turkey meat for slaughter were shown by Kondratowicz et al. [44]. The evaluation of sensory smell and colour of raw broiler chicken meat is, at the consumer level, of great significance since it is the most visible quality trait and, therefore, linked to consumer acceptance [27,28]. As reported by Katiyo et al. [2] for the consumer, odour is a more reliable indicator of spoilage of raw broiler chicken meat at retail and home than changes in appearance. The detection of an uncharacteristically pungent, fishy ammonia smell can be a warning signal to the consumer. Franke et al. [43] also reported that an unpleasant odour is a signal of microbiological spoilage in chicken meat. Colour may be a more distinguishing factor for red meat, such as beef, due to its relatively high myoglobin content [65].

## 4. Conclusions

To sum up, the study has indicated that by using the MALDI-TOF MS method, it is possible to assess the bacterial profile of poultry meat stored in conditions of stable, low refrigeration temperature maintained at 0.5 °C+/−0.5 °C. A detailed analysis of the dynamics of the development of individual bacterial species performed in this paper using the MALDI-TOF MS method is capable of contributing to expanding knowledge on the participation of individual bacterial species in the spoilage process of poultry meat stored in refrigerated conditions at a narrow, but stable level temperature range. The research and its findings should be treated as preliminary, paving the way to further research into optimising the chilling of poultry meat to extend its shelf life. However, further extensive research is necessary before relevant conclusions can be drawn regarding its application in the meat industry.

## Figures and Tables

**Table 1:** Specification of sensory characteristics adopted for raw meat from broiler chicken breasts stored refrigerated.

Score [CU]	Hedonic Scale	Sensory Descriptors			
		Smell	External Colour	Consistency	General Appearance
Desirability RANGE (3.51–5.0)	5	very conclusive, typical	balanced typical	elastic meat tissue, compact	faultless, moist surface, typical
	4	conclusive, typical	desired, less balanced, typical	rather elastic, when pressed, the deformation evens out	desirable, slightly dried surface
Adjusted desirability RANGE (2.51–3.5)	3	non-perceptible, slight change	rather desired, non-balanced, localised changes	permanent deformation on meat tissue when pressed	rather desirable, dried surface or slightly moist
Undesirable RANGE (1–2.5)	2	changed, light intensive, unacceptable	undesirable, localised changes, drips, yellow	meat tissue relaxed, flattens out after pressing	undesirable slimy, slightly sticky surface, locally changed colour
	1	changed, rotten	very undesirable, yellow or green in places	meat tissue relaxed after pressure, easily falls apart	very undesirable, surface sticky with mucus

**Table 2:** Results of the assessment of microbiological parameters of raw broiler chicken breast meat in refrigerated storage [Log CFU/g ± SD].

Time of Refrigerated Storage [d]	Total Count of Microorganisms	Number of Bacteria from:	
		Family Enterobacteriaceae	*Pseudomonas* Genera
1	2.34 ^a^ ± 0.19	<1.00 *	1.76 ^a^ ± 0.29
3	2.57 ^a^ ± 0.45	2.02 ^a^ ± 0.02	1.85 ^a^ ± 0.27
5	3.03 ^b^ ± 0.27	2.10 ^a^ ± 0.54	2.94 ^b^ ± 0.13
7	4.24 ^c^ ± 0.36	3.86 ^b^ ± 0.61	3.67 ^c^ ± 0.19
8	4.75 ^d^ ± 0.15	4.20 ^bc^ ± 0.13	4.70 ^d^ ± 0.59
9	5.06 ^d^ ± 0.22	4.45 ^bc^ ± 0.04	5.00 ^d^ ± 0.21
10	6.01 ^e^ ± 0.21	4.90 ^c^ ± 0.48	5.25 ^d^ ± 0.20
11	6.40 ^e^ ± 0.39	5.53 ^d^ ± 0.40	5.75 ^e^ ± 0.15
12 *p* Value	6.79 ^f^ ± 0.31 <0.0001	5.56 ^d^ ± 0.48 <0.0001	6.62 ^f^ ± 0.44 <0.0001

*—below the detection threshold [1 log CFU/g etc.]; ^a, b, c^ …—values in columns marked with different letters differ at *p* < 0.05.

**Table 3:** Isolated species from meat samples. “+”—occurrence of a bacterial isolate on the tested day.

Family	Genera	Species	Number of Isolates	Day								
				1	3	5	7	8	9	10	11	12
Moraxellaceae	*Acinetobacter*	*Acinetobacter johnsonii*	8	+	+	+	+					
Aeromonadaceae	*Aeromonas*	*Aeromonas bestiarum*	1	+								
Aeromonadaceae	*Aeromonas*	*Aeromonas eucrenophila*	1									+
Aeromonadaceae	*Aeromonas*	*Aeromonas molluscorum*	2			+	+					
Aeromonadaceae	*Aeromonas*	*Aeromonas popoffii*	2	+				+				
Aeromonadaceae	*Aeromonas*	*Aeromonas salmonicida*	6			+	+		+			
Aeromonadaceae	*Aeromonas*	*Aeromonas veronii*	2			+	+					
Bacillaceae	*Bacillus*	*Bacillus* spp.	2	+								
Enterobacteriaceae	*Buttiauxella*	*Buttiauxella agrestis*	2			+	+					
Enterobacteriaceae	*Buttiauxella*	*Buttiauxella gaviniae*	5		+			+	+	+		
Carnobacteriaceae	*Carnobacterium*	*Carnobacterium maltaromaticum*	2			+	+					
Enterobacteriaceae	*Citrobacter*	*Citrobacter braakii*	2			+	+					
Enterobacteriaceae	*Citrobacter*	*Citrobacter gillenii*	1			+						
Enterobacteriaceae	*Escherichia*	*Escherichia coli*	9	+	+	+	+	+				
Yersiniaceae	*Ewingella*	*Ewingella americana*	2			+	+					
Flavobacteriaceae	*Flavobacterium*	*Flavobacterium johnsoniae*	1	+								
Enterobacteriaceae	*Hafnia*	*Hafnia alvei*	6		+							+
Microbacteriaceae	*Microbacterium*	*Microbacterium liquefaciens*	8	+	+	+	+					
Microbacteriaceae	*Microbacterium*	*Microbacterium maritypicum*	2	+								
Micrococcaceae	*Micrococcus*	*Micrococcus luteus*	7	+	+							
Flavobacteriaceae	*Myroides*	*Myroides odoratimimus*	1		+							
Pseudomonadaceae	*Pseudomonas*	*Pseudomonas antarctica*	4	+	+							
Pseudomonadaceae	*Pseudomonas*	*Pseudomonas brassicacearum*	1						+			
Pseudomonadaceae	*Pseudomonas*	*Pseudomonas brenneri*	18	+		+	+	+	+		+	+
Pseudomonadaceae	*Pseudomonas*	*Pseudomonas caricapapayae*	2	+								
Pseudomonadaceae	*Pseudomonas*	*Pseudomonas cichorii*	2					+				
Pseudomonadaceae	*Pseudomonas*	*Pseudomonas congelans*	2			+	+					
Pseudomonadaceae	*Pseudomonas*	*Pseudomonas corrugata*	8	+	+	+	+	+				+
Pseudomonadaceae	*Pseudomonas*	*Pseudomonas extremorientalis*	3	+						+	+	
Pseudomonadaceae	*Pseudomonas*	*Pseudomonas flavescens*	4			+	+					
Pseudomonadaceae	*Pseudomonas*	*Pseudomonas fluorescens*	19	+	+	+	+		+	+	+	
Pseudomonadaceae	*Pseudomonas*	*Pseudomonas fragi*	132		+	+	+	+	+	+	+	+
Pseudomonadaceae	*Pseudomonas*	*Pseudomonas gessardii*	36	+	+	+	+	+	+	+	+	+
Pseudomonadaceae	*Pseudomonas*	*Pseudomonas chlororaphis*	2	+							+	
Pseudomonadaceae	*Pseudomonas*	*Pseudomonas kilonensis*	4			+	+					
Pseudomonadaceae	*Pseudomonas*	*Pseudomonas koreensis*	3	+	+							
Pseudomonadaceae	*Pseudomonas*	*Pseudomonas libanensis*	10	+		+	+	+	+	+	+	
Pseudomonadaceae	*Pseudomonas*	*Pseudomonas lundensis*	58		+	+	+	+	+	+	+	+
Pseudomonadaceae	*Pseudomonas*	*Pseudomonas monteilii*	2	+								
Pseudomonadaceae	*Pseudomonas*	*Pseudomonas orientalis*	1	+								
Pseudomonadaceae	*Pseudomonas*	*Pseudomonas oryzihabitans*	1		+							
Pseudomonadaceae	*Pseudomonas*	*Pseudomonas protegens*	2			+	+					
Pseudomonadaceae	*Pseudomonas*	*Pseudomonas proteolytica*	4	+					+	+		
Pseudomonadaceae	*Pseudomonas*	*Pseudomonas putida*	4	+	+							
Pseudomonadaceae	*Pseudomonas*	*Pseudomonas stutzeri*	1	+								
Pseudomonadaceae	*Pseudomonas*	*Pseudomonas synxantha*	4		+					+	+	
Pseudomonadaceae	*Pseudomonas*	*Pseudomonas taetrolens*	17			+	+	+	+	+	+	+
Pseudomonadaceae	*Pseudomonas*	*Pseudomonas tolaasii*	1	+								
Pseudomonadaceae	*Pseudomonas*	*Pseudomonas veronii*	7	+	+	+	+	+				
Moraxellaceae	*Psychrobacter*	*Psychrobacter spp.*	2	+								
Enterobacteriaceae	*Rahnella*	*Rahnella aquatilis*	2			+	+					
Nocardiaceae	*Rhodococcus*	*Rhodococcus erythropolis*	2		+							
Nocardiaceae	*Rhodotorula*	*Rhodotorula mucilaginosa*	7		+	+	+					
Micrococcaceae	*Rothia*	*Rothia nasimurium*	5		+	+	+					
Enterobacteriaceae	*Serratia*	*Serratia liquefaciens*	4		+	+	+					
Enterobacteriaceae	*Serratia*	*Serratia plymuthica*	1							+		
Enterobacteriaceae	*Serratia*	*Serratia proteamaculans*	6			+	+	+	+			+
Shewanellaceae	*Shewanella*	*Shewanella baltica*	4			+	+		+		+	
Staphylococcaceae	*Staphylococcus*	*Staphylococcus cohnii*	1		+							
Xanthomonadaceae	*Stenotrophomonas*	*Stenotrophomonas maltophilia*	3		+							
Streptococcaceae	*Streptococcus*	*Streptococcus pluranimalium*	1	+								
Yersiniaceae	*Yersinia*	*Yersinia enterocolitica*	4			+	+				+	+
Yersiniaceae	*Yersinia*	*Yersinia rohdei*	2					+		+		
**Total isolates**			**468**									

Explanation: “+”—occurrence of the bacterial isolate on the tested day.

**Table 4:** Results of the evaluation of sensory characteristics of raw broiler chicken meat in refrigerated storage (points).

Day of Refrigerated Storage [d]	Studied Traits			
	Smell	Colour	Consistency	General Appearance
1	4.96 ^a^ ± 0.20	4.98 ^a^ ± 0.14	5.00 ^a^ ± 0.00	5.00 ^a^ ± 0.00
3	4.68 ^a^ ± 0.49	4.94 ^a^ ± 0.24	4.96 ^a^ ± 0.20	4.95 ^a^ ± 0.22
5	4.46 ^ab^ ± 0.60	4.84 ^ab^ ± 0.39	4.65 ^ab^ ± 0.52	4.80 ^ab^ ± 0.45
7	4.01 ^b^ ± 0.22	4.10 ^ab^ ± 0.36	4.07 ^ab^ ± 0.33	4.09 ^bc^ ± 0.40
8	3.82 ^bc^ ± 0.44	3.92 ^bc^ ± 0.44	4.12 ^bc^ ± 0.41	3.82 ^c^ ± 0.54
9	3.15 ^c^ ± 0.48	3.48 ^c^ ± 0.50	3.50 ^c^ ± 0.50	3.35 ^c^ ± 0.48
10	2.48 ^d^ ± 0.17	3.14 ^cd^ ± 0.35	3.10 ^cd^ ± 0.30	3.12 ^cd^ ± 0.33
11	2.36 ^de^ ± 0.22	2.40 ^de^ ± 0.14	2.46 ^de^ ± 0.20	2.38 ^de^ ± 0.14
12	1.80 ^e^ ± 0.42	2.35 ^e^ ± 0.48	2.42 ^e^ ± 0.50	2.20 ^e^ ± 0.49
*p* Value	0.0001	0.0130	0.0217	0.0002

^a, b, c^…—Values in columns marked with different letters differ at *p* < 0.05.

## Data Availability

The data presented in this study are available on request from the corresponding author. The data are not publicly available due to privacy restrictions.

## Supplementary Materials

The following supporting information can be downloaded at: https://www.mdpi.com/article/10.3390/foods13244063/s1, Table S1. Correlation matrix on sensory analysis parameters and microbiological analysis results in broiler chicken breast meat during refrigerated storage.
