# Supplementary material for: Microbial and Sensory Quality Changes in Broiler Chicken Breast Meat During Refrigerated Storage
Source: Foods. 2024 Dec 17;13(24):4063. doi: 10.3390/foods13244063 (PMC11675927; doi:10.3390/foods13244063)
Supplement: Supplementary file 1 [file foods-13-04063-s001.zip › foods-3310497-supplementary.pdf]

Table S1. Correlation matrix on sensory analysis parameters and microbiological analysis results in broiler chicken breast meat during refrigerated storage.

Variable	Correlations (column stats) Marked correlation coefficients are significant with p < .05000 N=8 (Missing data were removed by chance)							
	Storage day	Smell	External colour	Consistency	General appearance	TVC	Enterobacteriaceae	*Pseudomonas* genera
Storage day	1.000000	-0.970333	-0.980637	-0.978115	-0.985806	0.991962	0.977435	0.992806
Smell	-0.970333	1.000000	0.994557	0.992411	0.990951	-0.973013	-0.936707	-0.968693
Colour	-0.980637	0.994557	1.000000	0.992646	0.997932	-0.987422	-0.966567	-0.973684
Consistency	-0.978115	0.992411	0.992646	1.000000	0.988589	-0.981631	-0.949032	-0.963047
General appearance	-0.985806	0.990951	0.997932	0.988589	1.000000	-0.987884	-0.971549	-0.981704
TVC	0.991962	-0.973013	-0.987422	-0.981631	-0.987884	1.000000	0.984033	0.979009
Enterobacteriaceae	0.977435	-0.936707	-0.966567	-0.949032	-0.971549	0.984033	1.000000	0.962160
Pseudomonas genera	0.992806	-0.968693	-0.973684	-0.963047	-0.981704	0.979009	0.962160	1.000000

Date / time / Temperature°C

23.11.2023 07:00 0.7

23.11.2023 07:15 0.7

23.11.2023 07:30 0.7

23.11.2023 07:45 0.7

23.11.2023 08:00 0.6

23.11.2023 08:15 0.6

23.11.2023 08:30 0.6

23.11.2023	08:45	0.5
23.11.2023	09:00	0.5
23.11.2023	09:15	0.5
23.11.2023	09:30	0.5
23.11.2023	09:45	0.5
23.11.2023	10:00	0.5
23.11.2023	10:15	0.4
23.11.2023	10:30	0.4
23.11.2023	10:45	0.4
23.11.2023	11:00	0.4
23.11.2023	11:15	0.5
23.11.2023	11:30	0.4
23.11.2023	11:45	0.4
23.11.2023	12:00	0.3
23.11.2023	12:15	0.3
23.11.2023	12:30	0.3
23.11.2023	12:45	0.3
23.11.2023	13:00	0.3
23.11.2023	13:15	0.3
23.11.2023	13:30	0.3

23.11.2023	13:45	0.4
23.11.2023	14:00	0.3
23.11.2023	14:15	0.3
23.11.2023	14:30	0.3
23.11.2023	14:45	0.4
23.11.2023	15:00	0.4
23.11.2023	15:15	0.4
23.11.2023	15:30	0.4
23.11.2023	15:45	0.3
23.11.2023	16:00	0.3
23.11.2023	16:15	0.4
23.11.2023	16:30	0.4
23.11.2023	16:45	0.4
23.11.2023	17:00	0.4
23.11.2023	17:15	0.3
23.11.2023	17:30	0.3
23.11.2023	17:45	0.4
23.11.2023	18:00	0.4
23.11.2023	18:15	0.4
23.11.2023	18:30	0.4

23.11.2023	18:45	0.5
23.11.2023	19:00	0.5
23.11.2023	19:15	0.5
23.11.2023	19:30	0.5
23.11.2023	19:45	0.5
23.11.2023	20:00	0.4
23.11.2023	20:15	0.5
23.11.2023	20:30	0.4
23.11.2023	20:45	0.4
23.11.2023	21:00	0.4
23.11.2023	21:15	0.4
23.11.2023	21:30	0.3
23.11.2023	21:45	0.3
23.11.2023	22:00	0.3
23.11.2023	22:15	0.3
23.11.2023	22:30	0.3
23.11.2023	22:45	0.3
23.11.2023	23:00	0.4
23.11.2023	23:15	0.4
23.11.2023	23:30	0.5

23.11.2023	23:45	0.5
23.11.2023	00:00	0.5
24.11.2023	00:15	0.5
24.11.2023	00:30	0.6
24.11.2023	00:45	0.6
24.11.2023	01:00	0.5
24.11.2023	01:15	0.6
24.11.2023	01:30	0.6
24.11.2023	01:45	0.6
24.11.2023	02:00	0.6
24.11.2023	02:15	0.6
24.11.2023	02:30	0.6
24.11.2023	02:45	0.6
24.11.2023	03:00	0.6
24.11.2023	03:15	0.6
24.11.2023	03:30	0.6
24.11.2023	03:45	0.6
24.11.2023	04:00	0.6
24.11.2023	04:15	0.6
24.11.2023	04:30	0.5

24.11.2023	04:45	0.5
24.11.2023	05:00	0.5
24.11.2023	05:15	0.5
24.11.2023	05:30	0.5
24.11.2023	05:45	0.5
24.11.2023	06:00	0.5
24.11.2023	06:15	0.5
24.11.2023	06:30	0.5
24.11.2023	06:45	0.5
24.11.2023	07:00	0.6
24.11.2023	07:15	0.6
24.11.2023	07:30	0.6
24.11.2023	07:45	0.6
24.11.2023	08:00	0.6
24.11.2023	08:15	0.5
24.11.2023	08:30	0.5
24.11.2023	08:45	0.5
24.11.2023	09:00	0.5
24.11.2023	09:15	0.5
24.11.2023	09:30	0.6

24.11.2023	09:45	0.6
24.11.2023	10:00	0.6
24.11.2023	10:15	0.7
24.11.2023	10:30	0.8
24.11.2023	10:45	0.9
24.11.2023	11:00	0.9
24.11.2023	11:15	1
24.11.2023	11:30	1
24.11.2023	11:45	1
24.11.2023	12:00	1
24.11.2023	12:15	1
24.11.2023	12:30	1
24.11.2023	12:45	1
24.11.2023	13:00	1
24.11.2023	13:15	1
24.11.2023	13:30	1
24.11.2023	13:45	1
24.11.2023	14:00	1
24.11.2023	14:15	1
24.11.2023	14:30	1

24.11.2023	14:45	1
24.11.2023	15:00	0.9
24.11.2023	15:15	0.9
24.11.2023	15:30	0.9
24.11.2023	15:45	0.9
24.11.2023	16:00	0.9
24.11.2023	16:15	0.9
24.11.2023	16:30	0.9
24.11.2023	16:45	0.9
24.11.2023	17:00	0.9
24.11.2023	17:15	0.9
24.11.2023	17:30	0.9
24.11.2023	17:45	0.9
24.11.2023	18:00	0.9
24.11.2023	18:15	0.9
24.11.2023	18:30	0.8
24.11.2023	18:45	0.8
24.11.2023	19:00	0.8
24.11.2023	19:15	0.8
24.11.2023	19:30	0.8

24.11.2023	19:45	0.8
24.11.2023	20:00	0.8
24.11.2023	20:15	0.8
24.11.2023	20:30	0.8
24.11.2023	20:45	0.8
24.11.2023	21:00	0.8
24.11.2023	21:15	0.8
24.11.2023	21:30	0.8
24.11.2023	21:45	0.8
24.11.2023	22:00	0.8
24.11.2023	22:15	0.8
24.11.2023	22:30	0.8
24.11.2023	22:45	0.8
24.11.2023	23:00	0.7
24.11.2023	23:15	0.7
24.11.2023	23:30	0.7
24.11.2023	23:45	0.7
25.11.2023	00:00	0.7
25.11.2023	00:15	0.7
25.11.2023	00:30	0.7

25.11.2023	00:45	0.7
25.11.2023	01:00	0.7
25.11.2023	01:15	0.7
25.11.2023	01:30	0.7
25.11.2023	01:45	0.7
25.11.2023	02:00	0.7
25.11.2023	02:15	0.7
25.11.2023	02:30	0.7
25.11.2023	02:45	0.7
25.11.2023	03:00	0.7
25.11.2023	03:15	0.7
25.11.2023	03:30	0.7
25.11.2023	03:45	0.7
25.11.2023	04:00	0.7
25.11.2023	04:15	0.7
25.11.2023	04:30	0.7
25.11.2023	04:45	0.7
25.11.2023	05:00	0.7
25.11.2023	05:15	0.7
25.11.2023	05:30	0.7

25.11.2023	05:45	0.7
25.11.2023	06:00	0.7
25.11.2023	06:15	0.8
25.11.2023	06:30	0.8
25.11.2023	06:45	0.8
25.11.2023	07:00	0.8
25.11.2023	07:15	0.8
25.11.2023	07:30	0.8
25.11.2023	07:45	0.8
25.11.2023	08:00	0.8
25.11.2023	08:15	0.8
25.11.2023	08:30	0.8
25.11.2023	08:45	0.8
25.11.2023	09:00	0.8
25.11.2023	09:15	0.7
25.11.2023	09:30	0.7
25.11.2023	09:45	0.7
25.11.2023	10:00	0.7
25.11.2023	10:15	0.7
25.11.2023	10:30	0.7

25.11.2023	10:45	0.6
25.11.2023	11:00	0.6
25.11.2023	11:15	0.6
25.11.2023	11:30	0.6
25.11.2023	11:45	0.5
25.11.2023	12:00	0.4
25.11.2023	12:15	0.4
25.11.2023	12:30	0.4
25.11.2023	12:45	0.4
25.11.2023	13:00	0.5
25.11.2023	13:15	0.5
25.11.2023	13:30	0.5
25.11.2023	13:45	0.5
25.11.2023	14:00	0.6
25.11.2023	14:15	0.6
25.11.2023	14:30	0.6
25.11.2023	14:45	0.7
25.11.2023	15:00	0.7
25.11.2023	15:15	0.8
25.11.2023	15:30	0.9

25.11.2023	15:45	1
25.11.2023	16:00	1
25.11.2023	16:15	1
25.11.2023	16:30	1
25.11.2023	16:45	1
25.11.2023	17:00	1
25.11.2023	17:15	1
25.11.2023	17:30	1
25.11.2023	17:45	1
25.11.2023	18:00	1
25.11.2023	18:15	0.9
25.11.2023	18:30	0.9
25.11.2023	18:45	0.9
25.11.2023	19:00	0.9
25.11.2023	19:15	0.9
25.11.2023	19:30	0.9
25.11.2023	19:45	0.9
25.11.2023	20:00	0.9
25.11.2023	20:15	0.9
25.11.2023	20:30	0.8

25.11.2023	20:45	0.8
25.11.2023	21:00	0.8
25.11.2023	21:15	0.8
25.11.2023	21:30	0.8
25.11.2023	21:45	0.8
25.11.2023	22:00	0.8
25.11.2023	22:15	0.8
25.11.2023	22:30	0.8
25.11.2023	22:45	0.8
25.11.2023	23:00	0.8
25.11.2023	23:15	0.8
25.11.2023	23:30	0.8
25.11.2023	23:45	0.8
26.11.2023	00:00	0.8
26.11.2023	00:15	0.8
26.11.2023	00:30	0.8
26.11.2023	00:45	0.8
26.11.2023	01:00	0.8
26.11.2023	01:15	0.8
26.11.2023	01:30	0.7

26.11.2023	01:45	0.7
26.11.2023	02:00	0.7
26.11.2023	02:15	0.7
26.11.2023	02:30	0.7
26.11.2023	02:45	0.6
26.11.2023	03:00	0.6
26.11.2023	03:15	0.6
26.11.2023	03:30	0.6
26.11.2023	03:45	0.6
26.11.2023	04:00	0.6
26.11.2023	04:15	0.6
26.11.2023	04:30	0.6
26.11.2023	04:45	0.6
26.11.2023	05:00	0.6
26.11.2023	05:15	0.6
26.11.2023	05:30	0.6
26.11.2023	05:45	0.6
26.11.2023	06:00	0.6
26.11.2023	06:15	0.6
26.11.2023	06:30	0.6

26.11.2023	06:45	0.7
26.11.2023	07:00	0.7
26.11.2023	07:15	0.7
26.11.2023	07:30	0.7
26.11.2023	07:45	0.7
26.11.2023	08:00	0.7
26.11.2023	08:15	0.7
26.11.2023	08:30	0.7
26.11.2023	08:45	0.7
26.11.2023	09:00	0.7
26.11.2023	09:15	0.7
26.11.2023	09:30	0.7
26.11.2023	09:45	0.8
26.11.2023	10:00	0.8
26.11.2023	10:15	0.8
26.11.2023	10:30	0.8
26.11.2023	10:45	0.8
26.11.2023	11:00	0.9
26.11.2023	11:15	0.9
26.11.2023	11:30	0.9

26.11.2023	11:45	0.9
26.11.2023	12:00	0.9
26.11.2023	12:15	0.9
26.11.2023	12:30	0.9
26.11.2023	12:45	0.9
26.11.2023	13:00	0.9
26.11.2023	13:15	0.9
26.11.2023	13:30	0.9
26.11.2023	13:45	0.9
26.11.2023	14:00	0.9
26.11.2023	14:15	0.9
26.11.2023	14:30	0.9
26.11.2023	14:45	0.9
26.11.2023	15:00	0.9
26.11.2023	15:15	0.9
26.11.2023	15:30	0.9
26.11.2023	15:45	0.9
26.11.2023	16:00	0.9
26.11.2023	16:15	0.9
26.11.2023	16:30	0.9

26.11.2023	16:45	0.9
26.11.2023	17:00	0.9
26.11.2023	17:15	0.9
26.11.2023	17:30	1
26.11.2023	17:45	1
26.11.2023	18:00	1
26.11.2023	18:15	1
26.11.2023	18:30	1
26.11.2023	18:45	1
26.11.2023	19:00	1
26.11.2023	19:15	1
26.11.2023	19:30	1
26.11.2023	19:45	1
26.11.2023	20:00	1
26.11.2023	20:15	1
26.11.2023	20:30	1
26.11.2023	20:45	1
26.11.2023	21:00	1
26.11.2023	21:15	1
26.11.2023	21:30	1

26.11.2023	21:45	0.9
26.11.2023	22:00	0.9
26.11.2023	22:15	0.8
26.11.2023	22:30	0.8
26.11.2023	22:45	0.8
26.11.2023	23:00	0.7
26.11.2023	23:15	0.7
26.11.2023	23:30	0.7
26.11.2023	23:45	0.7
27.11.2023	00:00	0.7
27.11.2023	00:15	0.7
27.11.2023	00:30	0.7
27.11.2023	00:45	0.7
27.11.2023	01:00	0.7
27.11.2023	01:15	0.7
27.11.2023	01:30	0.7
27.11.2023	01:45	0.7
27.11.2023	02:00	0.7
27.11.2023	02:15	0.7
27.11.2023	02:30	0.7

27.11.2023	02:45	0.7
27.11.2023	03:00	0.7
27.11.2023	03:15	0.7
27.11.2023	03:30	0.7
27.11.2023	03:45	0.7
27.11.2023	04:00	0.7
27.11.2023	04:15	0.7
27.11.2023	04:30	0.7
27.11.2023	04:45	0.7
27.11.2023	05:00	0.7
27.11.2023	05:15	0.7
27.11.2023	05:30	0.7
27.11.2023	05:45	0.7
27.11.2023	06:00	0.7
27.11.2023	06:15	0.7
27.11.2023	06:30	0.7
27.11.2023	06:45	0.7
27.11.2023	07:00	0.7
27.11.2023	07:15	0.7
27.11.2023	07:30	0.6

27.11.2023	07:45	0.6
27.11.2023	08:00	0.6
27.11.2023	08:15	0.6
27.11.2023	08:30	0.5
27.11.2023	08:45	0.5
27.11.2023	09:00	0.5
27.11.2023	09:15	0.5
27.11.2023	09:30	0.5
27.11.2023	09:45	0.5
27.11.2023	10:00	0.5
27.11.2023	10:15	0.5
27.11.2023	10:30	0.5
27.11.2023	10:45	0.5
27.11.2023	11:00	0.8
27.11.2023	11:15	0.8
27.11.2023	11:30	0.8
27.11.2023	11:45	0.8
27.11.2023	12:00	0.9
27.11.2023	12:15	0.9
27.11.2023	12:30	0.9

27.11.2023	12:45	0.9
27.11.2023	13:00	0.9
27.11.2023	13:15	0.9
27.11.2023	13:30	0.9
27.11.2023	13:45	0.9
27.11.2023	14:00	0.9
27.11.2023	14:15	0.9
27.11.2023	14:30	0.9
27.11.2023	14:45	0.8
27.11.2023	15:00	0.8
27.11.2023	15:15	0.8
27.11.2023	15:30	0.8
27.11.2023	15:45	0.7
27.11.2023	16:00	0.7
27.11.2023	16:15	0.7
27.11.2023	16:30	0.7
27.11.2023	16:45	0.7
27.11.2023	17:00	0.7
27.11.2023	17:15	0.7
27.11.2023	17:30	0.7

27.11.2023	17:45	0.7
27.11.2023	18:00	0.7
27.11.2023	18:15	0.6
27.11.2023	18:30	0.6
27.11.2023	18:45	0.6
27.11.2023	19:00	0.6
27.11.2023	19:15	0.6
27.11.2023	19:30	0.5
27.11.2023	19:45	0.5
27.11.2023	20:00	0.5
27.11.2023	20:15	0.5
27.11.2023	20:30	0.5
27.11.2023	20:45	0.4
27.11.2023	21:00	0.4
27.11.2023	21:15	0.4
27.11.2023	21:30	0.4
27.11.2023	21:45	0.4
27.11.2023	22:00	0.4
27.11.2023	22:15	0.5
27.11.2023	22:30	0.5

27.11.2023	22:45	0.4
27.11.2023	23:00	0.4
27.11.2023	23:15	0.4
27.11.2023	23:30	0.4
27.11.2023	23:45	0.3
28.11.2023	00:00	0.3
28.11.2023	00:15	0.3
28.11.2023	00:30	0.3
28.11.2023	00:45	0.3
28.11.2023	01:00	0.3
28.11.2023	01:15	0.3
28.11.2023	01:30	0.3
28.11.2023	01:45	0.3
28.11.2023	02:00	0.3
28.11.2023	02:15	0.4
28.11.2023	02:30	0.4
28.11.2023	02:45	0.4
28.11.2023	03:00	0.4
28.11.2023	03:15	0.4
28.11.2023	03:30	0.4

28.11.2023	03:45	0.4
28.11.2023	04:00	0.4
28.11.2023	04:15	0.5
28.11.2023	04:30	0.6
28.11.2023	04:45	0.6
28.11.2023	05:00	0.6
28.11.2023	05:15	0.6
28.11.2023	05:30	0.6
28.11.2023	05:45	0.6
28.11.2023	06:00	0.6
28.11.2023	06:15	0.6
28.11.2023	06:30	0.6
28.11.2023	06:45	0.7
28.11.2023	07:00	0.7
28.11.2023	07:15	0.7
28.11.2023	07:30	0.7
28.11.2023	07:45	0.8
28.11.2023	08:00	0.8
28.11.2023	08:15	0.8
28.11.2023	08:30	0.8

28.11.2023	08:45	0.8
28.11.2023	09:00	0.8
28.11.2023	09:15	0.8
28.11.2023	09:30	0.8
28.11.2023	09:45	0.9
28.11.2023	10:00	0.9
28.11.2023	10:15	0.9
28.11.2023	10:30	0.9
28.11.2023	10:45	0.9
28.11.2023	11:00	0.9
28.11.2023	11:15	0.9
28.11.2023	11:30	0.9
28.11.2023	11:45	0.9
28.11.2023	12:00	0.9
28.11.2023	12:15	0.9
28.11.2023	12:30	0.9
28.11.2023	12:45	1
28.11.2023	13:00	1
28.11.2023	13:15	1
28.11.2023	13:30	1

28.11.2023	13:45	1
28.11.2023	14:00	1
28.11.2023	14:15	1
28.11.2023	14:30	1
28.11.2023	14:45	1
28.11.2023	15:00	1
28.11.2023	15:15	1
28.11.2023	15:30	1
28.11.2023	15:45	1
28.11.2023	16:00	1
28.11.2023	16:15	1
28.11.2023	16:30	1
28.11.2023	16:45	1
28.11.2023	17:00	1
28.11.2023	17:15	1
28.11.2023	17:30	0.9
28.11.2023	17:45	0.9
28.11.2023	18:00	0.9
28.11.2023	18:15	0.9
28.11.2023	18:30	0.9

28.11.2023	18:45	0.9
28.11.2023	19:00	0.9
28.11.2023	19:15	0.9
28.11.2023	19:30	0.8
28.11.2023	19:45	0.8
28.11.2023	20:00	0.8
28.11.2023	20:15	0.8
28.11.2023	20:30	0.8
28.11.2023	20:45	0.8
28.11.2023	21:00	0.8
28.11.2023	21:15	0.7
28.11.2023	21:30	0.7
28.11.2023	21:45	0.7
28.11.2023	22:00	0.7
28.11.2023	22:15	0.7
28.11.2023	22:30	0.7
28.11.2023	22:45	0.7
28.11.2023	23:00	0.7
28.11.2023	23:15	0.7
28.11.2023	23:30	0.7

28.11.2023	23:45	0.7
29.11.2023	00:00	0.7
29.11.2023	00:15	0.7
29.11.2023	00:30	0.7
29.11.2023	00:45	0.7
29.11.2023	01:00	0.7
29.11.2023	01:15	0.7
29.11.2023	01:30	0.7
29.11.2023	01:45	0.7
29.11.2023	02:00	0.7
29.11.2023	02:15	0.7
29.11.2023	02:30	0.7
29.11.2023	02:45	0.7
29.11.2023	03:00	0.7
29.11.2023	03:15	0.7
29.11.2023	03:30	0.7
29.11.2023	03:45	0.7
29.11.2023	04:00	0.7
29.11.2023	04:15	0.7
29.11.2023	04:30	0.7

29.11.2023	04:45	0.7
29.11.2023	05:00	0.7
29.11.2023	05:15	0.7
29.11.2023	05:30	0.7
29.11.2023	05:45	0.7
29.11.2023	06:00	0.7
29.11.2023	06:15	0.7
29.11.2023	06:30	0.7
29.11.2023	06:45	0.8
29.11.2023	07:00	0.8
29.11.2023	07:15	0.8
29.11.2023	07:30	0.8
29.11.2023	07:45	0.8
29.11.2023	08:00	0.8
29.11.2023	08:15	0.8
29.11.2023	08:30	0.9
29.11.2023	08:45	0.9
29.11.2023	09:00	0.9
29.11.2023	09:15	0.9
29.11.2023	09:30	0.8

29.11.2023	09:45	0.8
29.11.2023	10:00	0.8
29.11.2023	10:15	0.7
29.11.2023	10:30	0.7
29.11.2023	10:45	0.7
29.11.2023	11:00	0.7
29.11.2023	11:15	0.6
29.11.2023	11:30	0.6
29.11.2023	11:45	0.6
29.11.2023	12:00	0.6
29.11.2023	12:15	0.6
29.11.2023	12:30	0.6
29.11.2023	12:45	0.6
29.11.2023	13:00	0.6
29.11.2023	13:15	0.5
29.11.2023	13:30	0.5
29.11.2023	13:45	0.5
29.11.2023	14:00	0.5
29.11.2023	14:15	0.5
29.11.2023	14:30	0.4

29.11.2023	14:45	0.4
29.11.2023	15:00	0.4
29.11.2023	15:15	0.4
29.11.2023	15:30	0.4
29.11.2023	15:45	0.5
29.11.2023	16:00	0.5
29.11.2023	16:15	0.5
29.11.2023	16:30	0.5
29.11.2023	16:45	0.5
29.11.2023	17:00	0.5
29.11.2023	17:15	0.5
29.11.2023	17:30	0.5
29.11.2023	17:45	0.5
29.11.2023	18:00	0.4
29.11.2023	18:15	0.4
29.11.2023	18:30	0.4
29.11.2023	18:45	0.4
29.11.2023	19:00	0.4
29.11.2023	19:15	0.4
29.11.2023	19:30	0.3

29.11.2023	19:45	0.3
29.11.2023	20:00	0.3
29.11.2023	20:15	0.3
29.11.2023	20:30	0.3
29.11.2023	20:45	0.3
29.11.2023	21:00	0.2
29.11.2023	21:15	0.2
29.11.2023	21:30	0.2
29.11.2023	21:45	0.3
29.11.2023	22:00	0.3
29.11.2023	22:15	0.3
29.11.2023	22:30	0.3
29.11.2023	22:45	0.3
29.11.2023	23:00	0.3
29.11.2023	23:15	0.3
29.11.2023	23:30	0.3
29.11.2023	23:45	0.3
30.11.2023	00:00	0.4
30.11.2023	00:15	0.4
30.11.2023	00:30	0.4

30.11.2023	00:45	0.4
30.11.2023	01:00	0.4
30.11.2023	01:15	0.5
30.11.2023	01:30	0.5
30.11.2023	01:45	0.5
30.11.2023	02:00	0.5
30.11.2023	02:15	0.5
30.11.2023	02:30	0.5
30.11.2023	02:45	0.5
30.11.2023	03:00	0.5
30.11.2023	03:15	0.5
30.11.2023	03:30	0.5
30.11.2023	03:45	0.5
30.11.2023	04:00	0.5
30.11.2023	04:15	0.5
30.11.2023	04:30	0.5
30.11.2023	04:45	0.5
30.11.2023	05:00	0.5
30.11.2023	05:15	0.5
30.11.2023	05:30	0.5

30.11.2023	05:45	0.5
30.11.2023	06:00	0.5
30.11.2023	06:15	0.5
30.11.2023	06:30	0.5
30.11.2023	06:45	0.6
30.11.2023	07:00	0.6
30.11.2023	07:15	0.6
30.11.2023	07:30	0.6
30.11.2023	07:45	0.6
30.11.2023	08:00	0.6
30.11.2023	08:15	0.6
30.11.2023	08:30	0.6
30.11.2023	08:45	0.6
30.11.2023	09:00	0.6
30.11.2023	09:15	0.7
30.11.2023	09:30	0.7
30.11.2023	09:45	0.7
30.11.2023	10:00	0.7
30.11.2023	10:15	0.7
30.11.2023	10:30	0.7

30.11.2023	10:45	0.7
30.11.2023	11:00	0.7
30.11.2023	11:15	0.7
30.11.2023	11:30	0.7
30.11.2023	11:45	0.7
30.11.2023	12:00	0.7
30.11.2023	12:15	0.8
30.11.2023	12:30	0.8
30.11.2023	12:45	0.8
30.11.2023	13:00	0.8
30.11.2023	13:15	0.8
30.11.2023	13:30	0.8
30.11.2023	13:45	0.8
30.11.2023	14:00	0.8
30.11.2023	14:15	0.8
30.11.2023	14:30	0.8
30.11.2023	14:45	0.8
30.11.2023	15:00	0.8
30.11.2023	15:15	0.8
30.11.2023	15:30	0.8

30.11.2023	15:45	0.8
30.11.2023	16:00	0.8
30.11.2023	16:15	0.8
30.11.2023	16:30	0.8
30.11.2023	16:45	0.7
30.11.2023	17:00	0.7
30.11.2023	17:15	0.7
30.11.2023	17:30	0.7
30.11.2023	17:45	0.7
30.11.2023	18:00	0.7
30.11.2023	18:15	0.7
30.11.2023	18:30	0.7
30.11.2023	18:45	0.7
30.11.2023	19:00	0.7
30.11.2023	19:15	0.7
30.11.2023	19:30	0.7
30.11.2023	19:45	0.6
30.11.2023	20:00	0.6
30.11.2023	20:15	0.6
30.11.2023	20:30	0.6

30.11.2023	20:45	0.6
30.11.2023	21:00	0.5
30.11.2023	21:15	0.5
30.11.2023	21:30	0.5
30.11.2023	21:45	0.5
30.11.2023	22:00	0.5
30.11.2023	22:15	0.5
30.11.2023	22:30	0.5
30.11.2023	22:45	0.5
30.11.2023	23:00	0.5
30.11.2023	23:15	0.5
30.11.2023	23:30	0.5
30.11.2023	23:45	0.5
01.12.2023	00:00	0.5
01.12.2023	00:15	0.5
01.12.2023	00:30	0.5
01.12.2023	00:45	0.5
01.12.2023	01:00	0.5
01.12.2023	01:15	0.5
01.12.2023	01:30	0.5

01.12.2023	01:45	0.5
01.12.2023	02:00	0.5
01.12.2023	02:15	0.5
01.12.2023	02:30	0.5
01.12.2023	02:45	0.5
01.12.2023	03:00	0.5
01.12.2023	03:15	0.6
01.12.2023	03:30	0.6
01.12.2023	03:45	0.6
01.12.2023	04:00	0.6
01.12.2023	04:15	0.6
01.12.2023	04:30	0.6
01.12.2023	04:45	0.6
01.12.2023	05:00	0.6
01.12.2023	05:15	0.6
01.12.2023	05:30	0.6
01.12.2023	05:45	0.6
01.12.2023	06:00	0.6
01.12.2023	06:15	0.7
01.12.2023	06:30	0.7

01.12.2023	06:45	0.7
01.12.2023	07:00	0.7
01.12.2023	07:15	0.7
01.12.2023	07:30	0.7
01.12.2023	07:45	0.7
01.12.2023	08:00	0.7
01.12.2023	08:15	0.7
01.12.2023	08:30	0.7
01.12.2023	08:45	0.7
01.12.2023	09:00	0.7
01.12.2023	09:15	0.7
01.12.2023	09:30	0.7
01.12.2023	09:45	0.7
01.12.2023	10:00	0.8
01.12.2023	10:15	0.8
01.12.2023	10:30	0.8
01.12.2023	10:45	0.8
01.12.2023	11:00	0.8
01.12.2023	11:15	0.8
01.12.2023	11:30	0.8

01.12.2023	11:45	0.8
01.12.2023	12:00	0.8
01.12.2023	12:15	0.8
01.12.2023	12:30	0.8
01.12.2023	12:45	0.8
01.12.2023	13:00	0.8
01.12.2023	13:15	0.8
01.12.2023	13:30	0.8
01.12.2023	13:45	0.8
01.12.2023	14:00	0.8
01.12.2023	14:15	0.8
01.12.2023	14:30	0.8
01.12.2023	14:45	0.9
01.12.2023	15:00	0.9
01.12.2023	15:15	0.9
01.12.2023	15:30	0.9
01.12.2023	15:45	1
01.12.2023	16:00	1
01.12.2023	16:15	1
01.12.2023	16:30	1

01.12.2023	16:45	1
01.12.2023	17:00	1
01.12.2023	17:15	1
01.12.2023	17:30	1
01.12.2023	17:45	1
01.12.2023	18:00	1
01.12.2023	18:15	1
01.12.2023	18:30	1
01.12.2023	18:45	0.9
01.12.2023	19:00	0.9
01.12.2023	19:15	0.9
01.12.2023	19:30	0.9
01.12.2023	19:45	0.9
01.12.2023	20:00	0.9
01.12.2023	20:15	0.9
01.12.2023	20:30	0.9
01.12.2023	20:45	0.8
01.12.2023	21:00	0.8
01.12.2023	21:15	0.8
01.12.2023	21:30	0.8

01.12.2023	21:45	0.8
01.12.2023	22:00	0.8
01.12.2023	22:15	0.8
01.12.2023	22:30	0.8
01.12.2023	22:45	0.8
01.12.2023	23:00	0.8
01.12.2023	23:15	0.7
01.12.2023	23:30	0.7
01.12.2023	23:45	0.7
02.12.2023	00:00	0.7
02.12.2023	00:15	0.7
02.12.2023	00:30	0.7
02.12.2023	00:45	0.7
02.12.2023	01:00	0.7
02.12.2023	01:15	0.7
02.12.2023	01:30	0.7
02.12.2023	01:45	0.7
02.12.2023	02:00	0.7
02.12.2023	02:15	0.7
02.12.2023	02:30	0.7

02.12.2023	02:45	0.7
02.12.2023	03:00	0.7
02.12.2023	03:15	0.7
02.12.2023	03:30	0.7
02.12.2023	03:45	0.7
02.12.2023	04:00	0.7
02.12.2023	04:15	0.7
02.12.2023	04:30	0.7
02.12.2023	04:45	0.7
02.12.2023	05:00	0.7
02.12.2023	05:15	0.7
02.12.2023	05:30	0.7
02.12.2023	05:45	0.7
02.12.2023	06:00	0.7
02.12.2023	06:15	0.7
02.12.2023	06:30	0.7
02.12.2023	06:45	0.7
02.12.2023	07:00	0.6
02.12.2023	07:15	0.6
02.12.2023	07:30	0.6

02.12.2023	07:45	0.6
02.12.2023	08:00	0.5
02.12.2023	08:15	0.5
02.12.2023	08:30	0.5
02.12.2023	08:45	0.5
02.12.2023	09:00	0.4
02.12.2023	09:15	0.4
02.12.2023	09:30	0.4
02.12.2023	09:45	0.4
02.12.2023	10:00	0.4
02.12.2023	10:15	0.4
02.12.2023	10:30	0.4
02.12.2023	10:45	0.4
02.12.2023	11:00	0.3
02.12.2023	11:15	0.3
02.12.2023	11:30	0.4
02.12.2023	11:45	0.4
02.12.2023	12:00	0.4
02.12.2023	12:15	0.4
02.12.2023	12:30	0.4

02.12.2023	12:45	0.5
02.12.2023	13:00	0.5
02.12.2023	13:15	0.5
02.12.2023	13:30	0.4
02.12.2023	13:45	0.4
02.12.2023	14:00	0.3
02.12.2023	14:15	0.3
02.12.2023	14:30	0.3
02.12.2023	14:45	0.4
02.12.2023	15:00	0.4
02.12.2023	15:15	0.4
02.12.2023	15:30	0.4
02.12.2023	15:45	0.4
02.12.2023	16:00	0.4
02.12.2023	16:15	0.5
02.12.2023	16:30	0.5
02.12.2023	16:45	0.5
02.12.2023	17:00	0.6
02.12.2023	17:15	0.6
02.12.2023	17:30	0.6

02.12.2023	17:45	0.6
02.12.2023	18:00	0.6
02.12.2023	18:15	0.7
02.12.2023	18:30	0.7
02.12.2023	18:45	0.7
02.12.2023	19:00	0.7
02.12.2023	19:15	0.7
02.12.2023	19:30	0.8
02.12.2023	19:45	0.8
02.12.2023	20:00	0.8
02.12.2023	20:15	0.8
02.12.2023	20:30	0.8
02.12.2023	20:45	0.8
02.12.2023	21:00	0.7
02.12.2023	21:15	0.7
02.12.2023	21:30	0.7
02.12.2023	21:45	0.7
02.12.2023	22:00	0.7
02.12.2023	22:15	0.7
02.12.2023	22:30	0.7

02.12.2023	22:45	0.7
02.12.2023	23:00	0.7
02.12.2023	23:15	0.7
02.12.2023	23:30	0.7
02.12.2023	23:45	0.7
03.12.2023	00:00	0.7
03.12.2023	00:15	0.6
03.12.2023	00:30	0.6
03.12.2023	00:45	0.6
03.12.2023	01:00	0.6
03.12.2023	01:15	0.6
03.12.2023	01:30	0.6
03.12.2023	01:45	0.6
03.12.2023	02:00	0.6
03.12.2023	02:15	0.6
03.12.2023	02:30	0.6
03.12.2023	02:45	0.6
03.12.2023	03:00	0.6
03.12.2023	03:15	0.6
03.12.2023	03:30	0.6

03.12.2023	03:45	0.6
03.12.2023	04:00	0.6
03.12.2023	04:15	0.6
03.12.2023	04:30	0.6
03.12.2023	04:45	0.6
03.12.2023	05:00	0.6
03.12.2023	05:15	0.6
03.12.2023	05:30	0.6
03.12.2023	05:45	0.6
03.12.2023	06:00	0.6
03.12.2023	06:15	0.6
03.12.2023	06:30	0.6
03.12.2023	06:45	0.6
03.12.2023	07:00	0.6
03.12.2023	07:15	0.6
03.12.2023	07:30	0.6
03.12.2023	07:45	0.5
03.12.2023	08:00	0.5
03.12.2023	08:15	0.5
03.12.2023	08:30	0.5

03.12.2023	08:45	0.6
03.12.2023	09:00	0.7
03.12.2023	09:15	0.7
03.12.2023	09:30	0.7
03.12.2023	09:45	0.7
03.12.2023	10:00	0.8
03.12.2023	10:15	0.8
03.12.2023	10:30	0.8
03.12.2023	10:45	0.8
03.12.2023	11:00	0.8
03.12.2023	11:15	0.8
03.12.2023	11:30	0.8
03.12.2023	11:45	0.8
03.12.2023	12:00	0.8
03.12.2023	12:15	0.8
03.12.2023	12:30	0.8
03.12.2023	12:45	0.8
03.12.2023	13:00	0.9
03.12.2023	13:15	0.9
03.12.2023	13:30	0.9

03.12.2023	13:45	0.9
03.12.2023	14:00	0.9
03.12.2023	14:15	0.9
03.12.2023	14:30	0.9
03.12.2023	14:45	0.9
03.12.2023	15:00	0.9
03.12.2023	15:15	0.9
03.12.2023	15:30	0.9
03.12.2023	15:45	0.9
03.12.2023	16:00	0.9
03.12.2023	16:15	0.9
03.12.2023	16:30	0.9
03.12.2023	16:45	0.9
03.12.2023	17:00	0.9
03.12.2023	17:15	0.9
03.12.2023	17:30	0.9
03.12.2023	17:45	0.9
03.12.2023	18:00	0.8
03.12.2023	18:15	0.8
03.12.2023	18:30	0.8

03.12.2023	18:45	0.8
03.12.2023	19:00	0.8
03.12.2023	19:15	0.8
03.12.2023	19:30	0.8
03.12.2023	19:45	0.8
03.12.2023	20:00	0.8
03.12.2023	20:15	0.8
03.12.2023	20:30	0.7
03.12.2023	20:45	0.7
03.12.2023	21:00	0.7
03.12.2023	21:15	0.7
03.12.2023	21:30	0.7
03.12.2023	21:45	0.7
03.12.2023	22:00	0.7
03.12.2023	22:15	0.7
03.12.2023	22:30	0.7
03.12.2023	22:45	0.7
03.12.2023	23:00	0.7
03.12.2023	23:15	0.7
03.12.2023	23:30	0.7

03.12.2023	23:45	0.7
04.12.2023	00:00	0.7
04.12.2023	00:15	0.7
04.12.2023	00:30	0.7
04.12.2023	00:45	0.7
04.12.2023	01:00	0.7
04.12.2023	01:15	0.7
04.12.2023	01:30	0.6
04.12.2023	01:45	0.6
04.12.2023	02:00	0.6
04.12.2023	02:15	0.6
04.12.2023	02:30	0.6
04.12.2023	02:45	0.6
04.12.2023	03:00	0.6
04.12.2023	03:15	0.6
04.12.2023	03:30	0.6
04.12.2023	03:45	0.6
04.12.2023	04:00	0.6
04.12.2023	04:15	0.6
04.12.2023	04:30	0.6

04.12.2023	04:45	0.
04.12.2023	05:00	0.6
04.12.2023	05:15	0.6
04.12.2023	05:30	0.6
04.12.2023	05:45	0.6
04.12.2023	06:00	0.6
04.12.2023	06:15	0.7
04.12.2023	06:30	0.7
04.12.2023	06:45	0.7
04.12.2023	07:00	0.8
04.12.2023	07:15	0.8
04.12.2023	07:30	0.8
04.12.2023	07:45	0.8
04.12.2023	08:00	0.8
04.12.2023	08:15	0.8
04.12.2023	08:30	0.8
04.12.2023	08:45	0.8
04.12.2023	09:00	0.8
04.12.2023	09:15	0.8
04.12.2023	09:30	0.8

04.12.2023	09:45	0.8
04.12.2023	10:00	0.9
04.12.2023	10:15	0.9
04.12.2023	10:30	0.9
04.12.2023	10:45	0.9
04.12.2023	11:00	0.9
04.12.2023	11:15	0.9
04.12.2023	11:30	0.9
04.12.2023	11:45	0.9
04.12.2023	12:00	1
04.12.2023	12:15	1
04.12.2023	12:30	1
04.12.2023	12:45	1
04.12.2023	13:00	1
04.12.2023	13:15	1
04.12.2023	13:30	1
04.12.2023	13:45	0.9
04.12.2023	14:00	0.9
04.12.2023	14:15	0.9
04.12.2023	14:30	0.9

04.12.2023	14:45	0.9
04.12.2023	15:00	0.9
04.12.2023	15:15	0.9
04.12.2023	15:30	0.9
04.12.2023	15:45	0.8
04.12.2023	16:00	0.8
04.12.2023	16:15	0.8
04.12.2023	16:30	0.8
04.12.2023	16:45	0.8
04.12.2023	17:00	0.8
04.12.2023	17:15	0.8
04.12.2023	17:30	0.8
04.12.2023	17:45	0.8
04.12.2023	18:00	0.8
04.12.2023	18:15	0.8
04.12.2023	18:30	0.7
04.12.2023	18:45	0.7
04.12.2023	19:00	0.7
04.12.2023	19:15	0.7
04.12.2023	19:30	0.7

04.12.2023	19:45	0.7
04.12.2023	20:00	0.6
04.12.2023	20:15	0.6
04.12.2023	20:30	0.6
04.12.2023	20:45	0.6
04.12.2023	21:00	0.6
04.12.2023	21:15	0.6
04.12.2023	21:30	0.6
04.12.2023	21:45	0.6
04.12.2023	22:00	0.6
04.12.2023	22:15	0.6
04.12.2023	22:30	0.6
04.12.2023	22:45	0.6
04.12.2023	23:00	0.6
04.12.2023	23:15	0.6
04.12.2023	23:30	0.5
04.12.2023	23:45	0.5
